# Retraction: MiR-520f promotes cell aggressiveness by regulating fibroblast growth factor 16 in hepatocellular carcinoma

**DOI:** 10.18632/oncotarget.28805

**Published:** 2025-12-31

**Authors:** Feng Feng Xu, Wen Feng Xie, Guo Qing Zha, Hong Wu Chen, Liang Deng

**Affiliations:** ^1^Department II of General Surgery, The Fifth Affiliated Hospital of Guangzhou Medical University, Guangzhou 510700, China; ^2^Department of Intensive Care Unit, The Eastern Hospital of the First Affiliated Hospital, Sun Yat-sen University, Guangzhou 510700, China; ^3^Upper Limb Department of Orthopedics, The Eastern Hospital of the First Affiliated Hospital, Sun Yat-sen University, Guangzhou 510700, China; ^4^Department of Emergency, The Eastern Hospital of the First Affiliated Hospital, Sun Yat-sen University, Guangzhou 510700, China; ^5^Department of Hepatobiliary Surgery, The Seventh Affiliated Hospital, Sun Yat-sen University, Shenzhen 518107, China; ^*^These authors have contributed equally to this work

**This article has been retracted:** Oncotarget has completed its investigation of this paper. Image forensic analysis confirmed that panel J of Figure 2 duplicates panel G of Figure 5, and that panel G of Figure 5 duplicates panel E of Figure 7. In addition, Figure 4C was found to duplicate Figure 5E in reference [1]. The authors agreed that the article should be retracted, although the journal has not received a formal retraction statement from them.

Original article: Oncotarget. 2017; 8:109546–109558. 109546-109558. https://doi.org/10.18632/oncotarget.22726
